# Supplementary material for: The interactome of intact mitochondria by cross-linking mass spectrometry provides evidence for coexisting respiratory supercomplexes
Source: Mol Cell Proteomics. 2017 Dec 8;17(2):216–32. doi: 10.1074/mcp.RA117.000470 (PMC5795388; doi:10.1074/mcp.RA117.000470)
Supplement: Supplemental Data [file supp_RA117.000470_133922_0_supp_23978_fzffwf.zip › spectra_annotation/mito_DR_spectra_annotation/341-1-4-1-20-1.pdf]

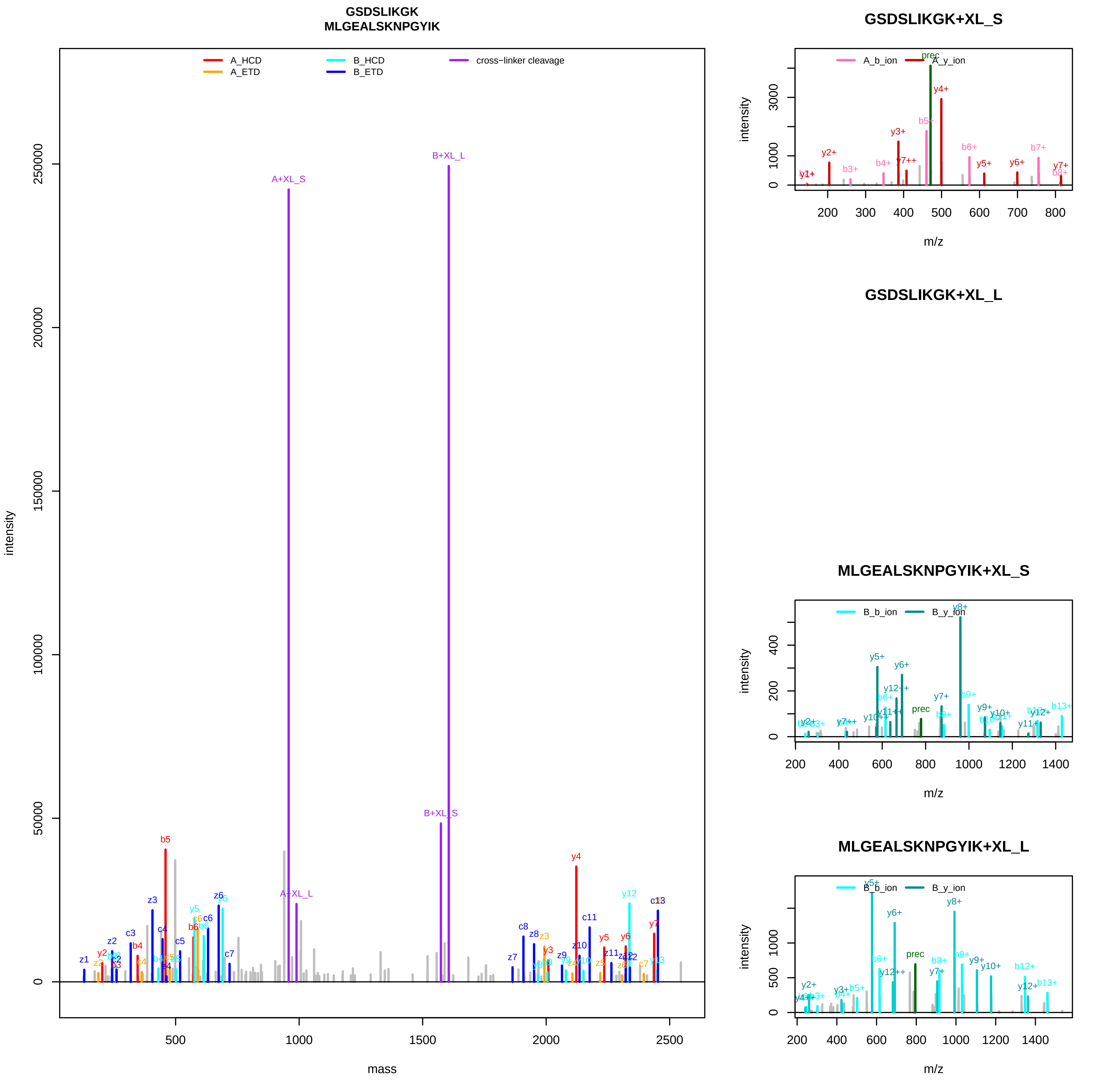

GSDSLIKGK
MLGEALSKNPGYIK
A_HCD
A_ETD
B_HCD
B_ETD
cross−linker cleavage
A+XL_S
B+XL_L
B+XL_S
A+XL_L
b5
y4
y12
c13
intensity
mass
0
50000
100000
150000
200000
250000
500
1000
1500
2000
2500
GSDSLIKGK+XL_S
A_b_ion
A_y_ion
prec
y4+
y3+
b5+
y2+
b3+
b4+
y7++
b6+
y5+
y6+
b7+
y7+
b8+
intensity
m/z
0
1000
3000
200
300
400
500
600
700
800
GSDSLIKGK+XL_L
MLGEALSKNPGYIK+XL_S
B_b_ion
B_y_ion
y8+
y5+
y6+
y12++
prec
y7+
b9+
b13+
intensity
m/z
0
200
400
200
400
600
800
1000
1200
1400
MLGEALSKNPGYIK+XL_L
B_b_ion
B_y_ion
y5+
y6+
y8+
prec
b8+
b9+
y9+
y10+
b12+
y12+
b13+
intensity
m/z
0
500
1000
200
400
600
800
1000
1200
1400
